# GRKs and β-Arrestins: “Gatekeepers” of Mitochondrial Function in the Failing Heart

**DOI:** 10.3389/fphar.2019.00064

**Published:** 2019-02-12

**Authors:** Daniela Sorriento, Jessica Gambardella, Antonella Fiordelisi, Guido Iaccarino, Maddalena Illario

**Affiliations:** ^1^Department of Advanced Biomedical Sciences, University of Naples Federico II, Naples, Italy; ^2^Health Innovation Unit, Campania Region, Naples, Italy

**Keywords:** G protein-coupled receptor kinase 2, β-arrestins, mitochondria, heart failure, cardiac damage

## Abstract

Mitochondrial regulation of energy production, calcium homeostasis, and cell death are critical for cardiac function. Accordingly, the structural and functional abnormalities of these organelles (mitochondrial dysfunction) contribute to developing cardiovascular diseases and heart failure. Therefore the preservation of mitochondrial integrity is essential for cardiac cell survival. Mitochondrial function is regulated by several proteins, including GRK2 and β-arrestins which act in a GPCR independent manner to orchestrate intracellular signaling associated with key mitochondrial processes. It is now ascertained that GRK2 is able to recover mitochondrial function in response to insults. β-arrestins affect several intracellular signaling pathways within the cell which in turn are involved in the regulation of mitochondrial function, but a direct regulation of mitochondria needs further investigations. In this review, we discuss the recent acquisitions on the role of GRK2 and β-arrestins in the regulation of mitochondrial function.

## Mitochondrial Functions in Damaged Heart

**FIGURE 1:**
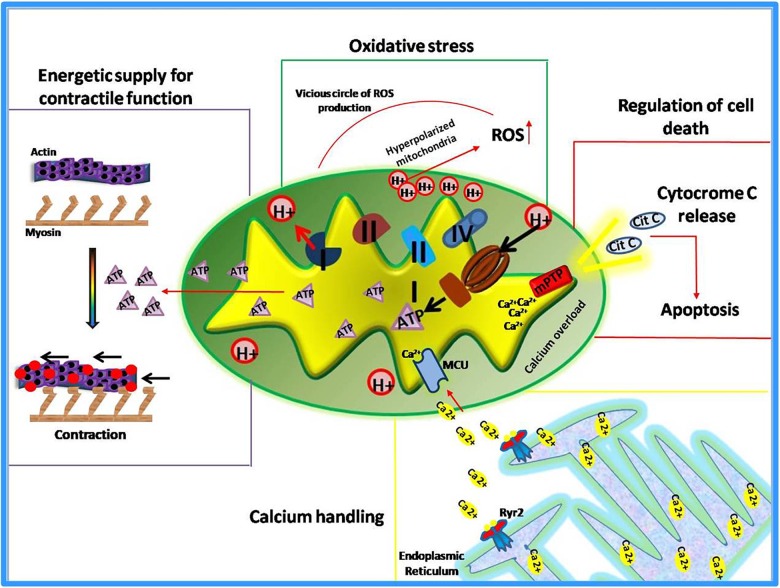
Energetic and non-energetic functions of mitochondria.

Known as “*powerhouse*” of the cell, mitochondria play essential roles in all human tissues, especially in those that are highly dependent on energy supply such as kidney, skeletal muscles, and myocardium. This latter, in particular, is the most metabolically active organ in the body. Its intense energy demand, needed to generate the contractile force, is supplied through the oxidative metabolism in mitochondria (Schaper et al., 1985; Barth et al., 1992; Hoppel et al., 2009). Therefore, it is not surprising that alterations of mitochondrial functions lead to the development of cardiac pathologies or susceptibility to injury. Indeed, mitochondrial dysfunction has been identified as the cause or a contributing factor in several heart diseases, thus several cardiac disorders, such heart failure, are currently defined “bioenergetic disease” (Murphy et al., 2016). However, beyond the regulation of energetic metabolism, mitochondria are now recognized to orchestrate multiple essential functions within the cell (Dorn, 2015). They sense smooth endoplasmic reticulum (ER) calcium release to modulate their metabolism and increase contractility (Chen et al., 2012; Dorn and Maack, 2013; Dorn, 2015). They are the main source of reactive oxygen species (ROS) to exert both physiological functions and pathological damage (Eisner et al., 2013). Moreover, mitochondria are the “gatekeepers” of programmed cell death (apoptosis and necrosis) (Wei et al., 2001; Whelan et al., 2012; Dorn, 2015). Also, these organelles are involved in the regulation of cardiomyocyte differentiation and embryonic cardiac development (Kasahara et al., 2013; Cho et al., 2014; Dorn, 2015). Thus, mitochondria exert both “energetic” and “non-energetic” functions (Figure 1) whose perturbations contribute to cardiac dysfunction (Rosenberg, 2004).

### Energetic Metabolism

Several pieces of evidence support the idea that heart failure is a “bioenergetic diseases” associated with a loss of energetic supply (Neubauer et al., 1997; Ingwall and Weiss, 2004). Indeed, by means of nuclear magnetic resonance (NMR), it has been shown that a significant reduction of cardiac ATP and phosphocreatine (PCr) content occurs in patients with heart failure (Starling et al., 1998; Beer et al., 2002). Experimental models of heart failure show that impaired energetic metabolism leads to heart damage (Ciccarelli et al., 2011). In physiological conditions, the myocardium is characterized by an intensive ATP intake and turnover (Balaban et al., 1986) that is regulated by phosphocreatine levels, produced by mitochondrial creatine kinase (Ingwall et al., 1985; Soboll et al., 1999). In basal conditions, two forms of this enzyme (active and inactive) are in dynamic equilibrium and when this equilibrium is altered, results in impaired control of respiratory chain activity and reduction of mitochondrial ATP production (Ashrafian, 2002). Moreover, a significant reduction of individual electron transport complexes I and IV has been described in humans and animal model of heart failure (Lemieux et al., 2011; Rosca et al., 2011),associated with a reduction of functional super-complexes (Rosca et al., 2008). Defects in the mitochondrial respiratory chain have been observed both in early stage and end-stage of chronic heart failure (Lemieux et al., 2011). All these evidence confirm the involvement of impaired energetic metabolism in the development and progression of cardiac disease.

### Mitochondrial ROS Production

Mitochondrial respiration through the electron transport chain (ETC) activity drives ATP synthesis and ROS production. In a healthy heart, the generation of superoxide radical is effectively neutralized by superoxide dismutase (Boveris et al., 1976). MnSOD silencing, the main mitochondrial antioxidant enzyme, is known to produce dilated cardiomyopathy leading to early postnatal death (Fukai and Ushio-Fukai, 2011). When mitochondrial respiration is compromised, decreased ATP production and increased oxidative stress occur (Lesnefsky et al., 2001). Elevated levels of ROS are able to induce oxidative modifications of specific mitochondrial proteins (Complex I and II, and Aconitase) that further stimulate ROS production in a vicious circle (Chen and Zweier, 2014). Moreover, a compromised antioxidant capacity also contributes to mitochondrial-mediated oxidative stress. The high levels of mitochondrial ROS induce alterations of main signaling pathways through oxidative modifications of important proteins such as cardiac ryanodine receptor (RyR2); indeed, the oxidation of this receptor alters its conformation and function contributing to the development of arrhythmias or heart failure (Oda et al., 2015).

### Mitochondrial Dependent Apoptosis

It is ascertained that mitochondria, the primary sensor of metabolic stress, activate the programmed cell death. The mitochondria-dependent activation of apoptotic processes occurs in response to irreversible damage induced by intense or perpetuate metabolic stress (i.e., during acute or chronic ischemia) (Chistiakov et al., 2018). Such a condition increases mitochondrial permeability leading to cytochrome c release from mitochondria to the cytosol thus activating apoptosis (Karbowski et al., 2002). Besides this classical pathway, there are also other mechanisms by which mitochondria activates cell death in the failing heart (Dorn and Kirshenbaum, 2008; Dorn, 2010). During cardiac ischemia, a mitochondrial serine protease, known as high-temperature requirement A2 (HtrA2), is also released from mitochondria and promotes caspase activation and apoptosis (Dorn and Kirshenbaum, 2008; Piquereau et al., 2013). Several pieces of evidence suggest that abnormal cardiomyocyte apoptosis also occurs in both animal models and humans with arterial hypertension. Under this pathological stress, a functional cross-talk between death receptors pathways and mitochondrial-dependent apoptosis has been described, that is mediated by the Bcl2 family (Gonzalez et al., 2003). In addition, alterations of the mitochondrial apoptotic pathway have been described in both hypertrophic and dilative cardiomyopathy (Harvey and Leinwand, 2011). Overall the loss of cardiomyocytes due to apoptosis dysregulation is detrimental to cardiac function, and all recent studies support the key role of mitochondria in such phenomenon.

### Mitochondrial Dynamics and Turnover

Mitochondrial fusion, fission, and trafficking, collectively called “mitochondrial dynamics”, are the regulators of Mitochondrial Quality Control, an essential process that preserves mitochondrial function and ensures cell survival (Ni et al., 2015; Shirihai et al., 2015; Sorriento et al., 2017). This process includes several mechanisms (Shirihai et al., 2015):

(a) fission/fusion, that allows segregation of damaged mitochondria;
(b) mitophagy, that removes the irreversibly damaged mitochondria;
(c) mitochondrial biogenesis, that ensures the generation of new intact mitochondria and new mtDNA molecules.

Perturbations of one or more of these mechanisms culminate in altered mitochondrial architecture and function. In failing heart, structural changes in mitochondria have been frequently observed, including giant mitochondria due to excessive mitochondrial fusion (Tandler et al., 2002). The balanced equilibrium between fusion and fission is switched toward fission during ischemia. Indeed, the up-regulation of dynamin-related protein 1 (Drp1), a key activator of mitochondrial fission, induces prominent mitochondrial fragmentation that in turn leads to loss of mitochondrial potential and apoptosis (Karbowski et al., 2002; Ong et al., 2010). Moreover, alterations in mitochondrial fragmentation or hyperplasia resulting from compromised fission/fusion balance have also been described in human and animal model of heart failure (Sabbah et al., 1992; Goh et al., 2016). The irreversibly damaged mitochondria are cleared in the cardiac cells by effective mitophagy, a tightly regulated process. In a healthy heart, PINK1 localizes on mitochondrial surface promoting parkin-dependent ubiquitination of MFN2 with the recruitment of several autophagy adaptors and formation of autophagosome around damaged mitochondria (Karbowski et al., 2002). Alterations in this pathway have been described in several cardiac diseases such as ischemic heart disease, cardiac hypertrophy, heart failure and dilated cardiomyopathy (Chistiakov et al., 2018). The mechanisms that lead to alterations in mitochondria clearance are not completely understood. Likely, an excessive autophagy occurs during acute cardiac injury leading also to the loss of functional organelles. On the contrary, mitophagy flux is reduced during the late stages of cardiac diseases thus promoting accumulation of damaged mitochondria, severe oxidative stress and cardiomyocytes apoptosis (Campos et al., 2016). Indeed, a reduction in autophagic activity is associated with poor prognosis of patients with heart disease (Campos et al., 2016). Beside the management of pre-existent mitochondria, also the de-novo synthesis of these organelles seems to be compromised in failing hearts. Replication of mtDNA is significantly impaired in heart failure resulting in depletion of mt-DNA-encoded proteins and in altered mitochondrial biogenesis (Karamanlidis et al., 2014). All these pieces of evidence suggest that mitochondrial turnover is a common compromised process in cardiac dysfunctions, pointing to another aspect of mitochondrial biology with a key role in cardiomyocyte function and survival.

## Grks and β-Arrestins: the Non-Gpcr Signaling

G Protein-CoupledReceptor (GPCR) Kinase (GRKs) and β-arrestins are key regulators of GPCR signaling (Ferguson, 2001; Kohout and Lefkowitz, 2003; Santulli and Iaccarino, 2016; Sorriento et al., 2016). Indeed, GRKs are recruited to the plasma membrane when the receptor is activated by agonist binding. Here, GRKs phosphorylate GPCRs favoring the recruitment of β-arrestin which in turn promotes rapid receptors desensitization or their clathrin-mediated endocytosis and internalization (Ferguson et al., 1996; Goodman et al., 1996; Ferguson, 2001; Pierce et al., 2002; Lefkowitz and Shenoy, 2005). Several evidence endorse the proof of concept that GRKs and β-arrestins also regulate intracellular signaling independently from GPCR by affecting non-GPCR receptors or by direct interaction with target molecules (Kang et al., 2005; Ma and Pei, 2007; Penela et al., 2010; Evron et al., 2012). Among GRKs, GRK2 is emerging as a key node in signal transduction pathways, displaying a very complex “interactome” (Penela et al., 2010). β-arrestins also function as scaffold proteins that interact with several cytoplasmic molecules (DeFea et al., 2000; McDonald et al., 2000) or interact in the cytosol with regulators of transcription factors, such as IκBαand MDM2,to regulate transcription indirectly (Wang et al., 2003; Luan et al., 2005). Thus, these molecules are able to regulate several key processes within the cell in a GPCR-independent manner, affecting cell biology both in physiological and pathological conditions.

The functional cross-talk between mitochondria and other cellular compartments has been shown to regulate mitochondrial function (Hajnoczky et al., 2000; Galganska et al., 2010; Viola and Hool, 2010; Li et al., 2018). This interplay has a key physiological role, placing the mitochondria under the regulation of extracellular stimuli and thus ensuring the adaptation of mitochondrial activity to environmental needs. This functional cross-talk is regulated through the activity of several proteins, among whichGRK2 and β-arrestins are potential candidates. Indeed, these molecules are able to move among different compartments and interact with different partners thus interfering with the mitochondrial signaling transduction pathway. GRK2 and β-arrestins regulate mitochondrial function in cardiac cells through mechanisms which are independent of GPCR signaling. This issue was only recently investigated but several data have already been generated with important translational implications.

### GRK2: A Dynamic and Multifunction Protein

GRKs are critical regulators of cardiac function both in physiological and pathological conditions. Among them, GRK2 and GRK5 are the most abundant G protein-coupled receptor kinases in the heart. In particular, GRK2 is essential for cardiac health. Indeed, the ablation of GRK2 gene in myocytes affects cardiac phenotypes in adulthood leading to a prevalent eccentric remodeling after chronic exposure to β adrenergic stimulation (Matkovich et al., 2006; Sorriento et al., 2015). Moreover, the total deletion of the kinase is lethal by preventing the correct development of the cardiovascular system in prenatal life (Raake et al., 2012). This developmental importance of GRK2 also concerns the endothelium. Indeed, the deletion of GRK2 in endothelial cells resulted in alteration of vascular phenotype and integrity, due to an increase of inflammation and oxidative stress (Ciccarelli et al., 2013; Rivas et al., 2013). Conversely, the deletion of GRK5 does not affect heart function but even ameliorates cardiac responses to insults (Hullmann et al., 2014). These data suggest that even though these kinases are both involved in the regulation of cardiac biology, GRK2 is the one fundamental for cardiac cell survival both in physiological and pathological conditions. It is clear that this effect could not be limited to the regulation of GPCR activity on plasma membrane where kinase effects seem, on the contrary, to trigger and sustain the development of heart failure (Lymperopoulos et al., 2012). That’s the reason why in the last decade research focused on the identification of other kinase activities within the cell which were independent of GPCR. A previous report from Mayor group clearly summarizes the novel identified GRK2 substrates and their functional roles in several conditions (cardiovascular diseases, inflammation, cancer) (Ribas et al., 2007; Penela et al., 2010). GRK2 has been shown to associate with PI3K, GIT, caveolin, MEK, AKT, α-actinin, clathrin, calmodulin, c-SRC, PKA, PKC, IkBα, and RKIP, regulating different signal transduction pathways within the cell (Ribas et al., 2007; Sorriento et al., 2015). Moreover, GRK2 can also interact with non–GPCR receptors, such as Ins-R, PDGF-R, and EGF-R (Hupfeld and Olefsky, 2007; Cipolletta et al., 2009).

Based on these findings, the idea that GRK2 is a dynamic molecule, that moves within the cell depending on cell requirements, started to take ground (Sorriento et al., 2014; Sorriento et al., 2016) suggesting that the regulation of GRK2 trafficking within the cell could be a potential strategy to regulate the adaptative effects of the kinase on cell functions (Sorriento et al., 2014).

### β-Arrestins and the Signalosomes

Besides their cardioprotective role through means of GPCR desensitization, common acquisitions suggest that β-arrestins also function as GPCR signal transducers (Luttrell and Lefkowitz, 2002). They directly initiate signaling through the formation of multiprotein signaling complexes, known as a “signalosomes,” in which they act as scaffolds, adaptors, and signal transduction proteins (Lefkowitz et al., 2006). They can form complexes with several signaling proteins, including Src family tyrosine kinases and components of the ERK1/2 and JNK3 MAP kinase cascades (DeWire et al., 2007; Kang et al., 2014). Moreover, β-arrestins can also regulate gene transcription in the nucleus. Indeed, they are able to interact with IkBα and sequester the complex IkBα-NFkB in the cytosol, thus inhibiting NFkB transcription activity (Witherow et al., 2004). More than 300 proteins have been identified that interact with β-arrestins with multiple implications in most key processes within the cell (Xiao et al., 2007). These proteins are mainly localized in the cytosol but they are also distributed in other compartments including mitochondria.

## Grk2 Dependent Regulation of Mitochondrial Function

### Energetic Metabolism and Mitochondrial Dynamics

Among the novel functions of GRK2, in the last decade, the potential role of the kinase in the regulation of the metabolic state of the cell emerged (Cipolletta et al., 2009; Sorriento et al., 2014). The evidence comes from the demonstration that GRK2 accumulation leads to the shut-off of insulin signaling and inhibits glucose extraction (Usui et al., 2004; Cipolletta et al., 2009; Ciccarelli et al., 2011). In the cardiovascular setting, given the key role of mitochondria to supply the energy need of the heart and the importance of GRK2 for cardiac biology, research also focused on the identification of a potential role of GRK2 in the regulation of energy metabolism leading to the discovery of GRK2as key mediator of production and expenditure of energy within the cell (Fusco et al., 2012; Chen et al., 2013). The first evidence of GRK2 localization at mitochondria comes from the study of Obrenovich’s group in a rat model of Alzheimer disease (Obrenovich et al., 2006). In the early pathogenesis of Alzheimer Disease and in ischemia-reperfusion brain injury models, GRK2 accumulates in damaged mitochondria, suggesting a role for the kinase in this organelle. However, Fusco and colleagues were the first who effectively identified the kinase role in mitochondria (Fusco et al., 2012). Indeed, they showed that the overexpression of this kinase increased mitochondrial mass, ATP production and mitochondrial biogenesis (Fusco et al., 2012). Ischemia causes acute accumulation of GRK2 in mitochondria both *in vitro* and *in vivo*, and reperfusion reverted such effect. The overexpression of the kinase protects ATP production even after hypoxia/reperfusion damage (Fusco et al., 2012). This suggests a potential role of the kinase in energy production, which is particularly relevant for tissues that need a great amount of energy to work well, such as the heart. Successively, other reports confirmed that GRK2 localizes into mitochondria. Indeed, Chen showed that both in hearts *in vivo* and in cultured myocytes, GRK2 localizes into mitochondria after an ischemia-reperfusion insult. The authors also propose a potential mechanism by which the kinase is able to traffic to mitochondria (Chen et al., 2013). In particular, they demonstrate that phosphorylation at residue Ser670 within the carboxyl-terminus of GRK2 by extracellular signal-regulated kinase (ERK) allows GRK2 to bind the heat shock protein 90 (HSP90), which chaperones the kinase to mitochondria (Chen et al., 2013). Accordingly, the same Authors also show that a mutant form of GRK2, that cannot bind HSP90, does not localize to mitochondria (Sato et al., 2018). Mitochondrial localization of the kinase is not limited to cardiac cells. Indeed, we demonstrated that GRK2 localizes into mitochondria of macrophagic cells in a time-dependent manner and an early translocation supports the cell to better respond to LPS dependent mitochondrial dysfunction (Sorriento et al., 2013). In these cells, the overexpression of the carboxy-terminal domain of GRK2 (βARK-ct), known to displace GRK2 from plasma membranes, induces earlier localization of GRK2 to mitochondria in response to LPS leading to increased mt-DNA transcription and reduced ROS production and cytokine expression (Sorriento et al., 2013). These data confirm that the mitochondrial localization of GRK2 ameliorates mitochondrial function, as shown in other models (Fusco et al., 2012). Accordingly, Franco recently showed that the overexpression of GRK2 protects mitochondria from the damage induced by ionizing radiation. Indeed, GRK2 favors the rescue of mitochondrial mass, morphology, and respiration (Franco et al., 2018). On the opposite, the kinase deletion accelerates degenerative processes induced by the exposure to ionizing radiation. This evidence clearly supports the idea that GRK2 is beneficial for mitochondrial function and is effective to protect mitochondria from insults. The mechanism involves a novel “interactome” of GRK2 which includes HSP90, as also previously demonstrated (Chen et al., 2013), and molecules involved in the regulation of mitochondrial dynamics, mitofusins (MFN-1 and MFN-2) (Franco et al., 2018). GRK2 dynamically binds MFN-1/2 by means of HSP90 and phosphorylates these molecules affecting mitochondrial fusion (Franco et al., 2018). MFN-1 and 2 are key regulators of mitochondrial fusion and fission processes that are critical for cardiac health (Santel, 2006). Recently, it has been demonstrated that these molecules can adopt either a fusion-constrained or a fusion-permissive molecular conformation that allows them to regulate mitochondrial dynamics (Franco et al., 2016). The imbalance between fission and fusion causes mitochondrial dysfunction. The finding that GRK2 is able to phosphorylate and activate these molecules suggest its involvement in mitochondrial dynamics and biology. This is the first finding regarding a phosphorylation-dependent regulation of the activity of the mitofusins. Likely, GRK2 by phosphorylating mitofusins can orchestrate mitochondrial dynamics. However, further data are needed to support this hypothesis.

### Apoptosis

Even though all *in vitro* findings strongly support a protective effect of GRK2 in damaged mitochondria, other reports, on the contrary, suggest a pro-death role of the kinase in this organelle. Indeed, in cardiac myocytes, the inhibition of GRK2 increased ATP production whereas the overexpression of the kinase increased oxidative stress and negatively regulated FA oxidation (Sato et al., 2015). Moreover, mitochondrial GRK2 is reported to promote cell death in ischemic myocytes and its inhibition by means of βARKct is reported to be cardioprotective.(Chen et al., 2013; Woodall et al., 2014). Conversely, the same authors previously published that this pro-apoptotic effect of GRK2 was only due to its effects on plasma membrane through the regulation of AKT signaling, also showing that the treatment with βARK-ct inhibited such phenomenon (Brinks et al., 2010). Thus, it is not clear yet whether the effects of βARK-ct could depend on GRK2 reduction in mitochondria or in the plasma membrane. Further studies are needed to better clarify this issue.

Also, the same Authors recently show that a mutant form of GRK2, that cannot bind HSP90, inhibits kinase localization in mitochondria and confers protection in response to ischemia-reperfusion (Sato et al., 2018).

In this context, the disruption of GRK2 binding with HSP90 has been shown to decrease the expression of endogenous GRK2 in a dose- and time-dependent manner (Luo and Benovic, 2003) and this could justify the cardioprotective role of βARKct and of GRK2 mutant. Given this evidence, it was expected that both βARKct and the expression of the mutant form of GRK2 would cause the total reduction of the kinase within the cell. Actually, total levels of GRK2 were not modified in both Chen’s and Sato’s studies which are therefore in contrast with literature. Given these discrepancies, the exact role of mitochondrial GRK2 in apoptotic processes still remains to be clarified.

### GRK2 in Mitochondria: Detrimental or Cardioprotective?

Actually, contrasting evidence exists on the role of GRK2 in mitochondria. To reconcile these opposing findings, several considerations are to be taken into account.

(a) Membrane displacement of GRK2 from the plasma membrane by means of βARKct overexpression does not mean lack of kinase activity. βARKct reduces GRK2 levels on the plasma membrane but also induces its localization in other cellular compartments, such as mitochondria (Sorriento et al., 2013). Giving this notion, the cardioprotective effect of βARKct in ischemic myocytes is in agreement with kinase accumulation in mitochondria.
(b) The overexpression is a complex maneuver that drastically upsets cell biology. Indeed, GRK2 overexpression increased kinase levels in all cellular compartments, including plasma membranes, whereGRK2induces apoptotic events by affecting GPCR signaling. Indeed, GRK2 induces oxidative stress and apoptosis in cardiac myocytes in the same manner of beta-adrenergic receptor stimulation, and kinase inhibition prevents such events (Theccanat et al., 2016). Thus, GRK2 dependent induction of apoptosis depends on its levels of expression on membranes being limited to its effects on GPCR signaling independently from mitochondria.
(c) Moreover, it should be considered that in response to insults the cell activates protective mechanisms to avoid irreversible damage. Mitochondrial fission, for instance, is activated to better respond to stress conditions by eliminating damaged mitochondria and restoring the normal cell activity. The apoptosis is a programmed cell death that is induced when cell damage is irreversible. Therefore, GRK2 dependent cell death could not be strictly dependent on a deleterious action of the kinase but would rather be the result of an advanced and irreversible mitochondrial damage that GRK2 is not able to stop.
(d) Timing is also a critical point to take into account. Indeed, the protective role of GRK2 has been shown in response to acute insults but the chronic responses in animal models could be different and negatively affect mitochondrial biology due to continuous activation of intracellular signaling.

**FIGURE 2:**
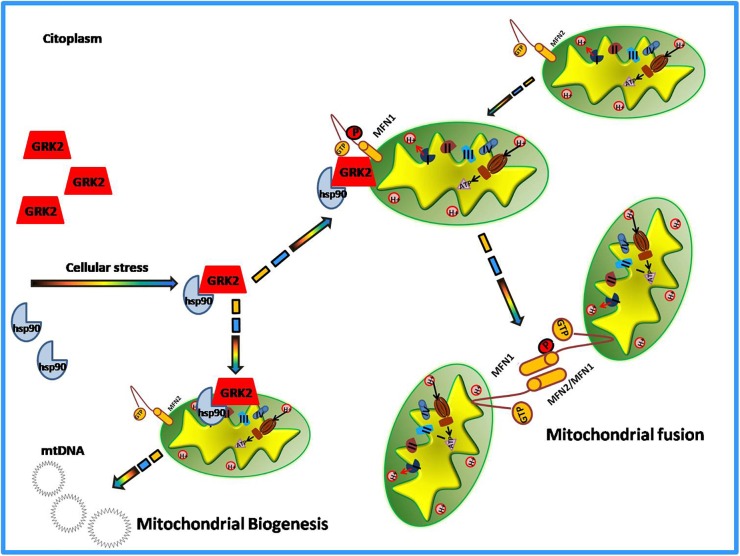
GRK2 activities in mitochondria.

Thus, altogether these findings support the proof of concept that GRK2 is a dynamic and multifunction protein whose actions in mitochondria likely aims to protect the cell from irreversible damage due to external insults (Figure 2). This activity is especially significant in those conditions, such as heart failure, characterized by remarkable mitochondrial dysfunction. Thus, the possibility to specifically deliver the kinase to mitochondria (i.e., through means of plasma membrane displacement such as βARK-ct) appears a promising strategy to recover mitochondrial function in damaged cardiac cells.

## β-Arrestins and Mitochondrial Activity

Among the numerous actions of β-arrestins within the cell, growing evidence suggests their involvement in the regulation of mitochondrial function in the heart. In particular, it has been shown that such proteins interfere with key mitochondrial processes such as cell death, ROS production, and respiration. The involvement of β-arrestins in the regulation of apoptotic processes is very controversial. Indeed, it has been suggested that these proteins can both promote and inhibit cell death probably due to different stimuli and cell type. In Mouse Embryonic Fibroblasts (MEFs), in response to apoptotic stimuli, β-arrestins are cleaved by multiple caspases at Asp380 generating a small fragment of 380 amino acids (Kook et al., 2014). This latter translocates to mitochondria and cooperates with tBID to induce the release of cytochrome C and consequently cell death (Youle and Strasser, 2008; Galluzzi et al., 2012). Conversely, in response to IGF-1 stimulation,β-arrestins mediate the activation of PI3K with subsequent activation of AKT (Gu et al., 2015). The PI3-kinase/AKT signaling pathway blocks caspase activity and consequently apoptosis (Kennedy et al., 1997). Also, in response to oxidative stressβ-arrestins exert an anti-apoptotic effect. Indeed, in HEK-293 cells stimulated with H2O2 β-arrestins bind the C-terminal domain of the Apoptosis Signal-regulating Kinase 1 (ASK1) inducing its ubiquitination and degradation by the proteasome (Zhang et al., 2009). Giving these opposing findings, further data are needed to better clarify the involvement of β-arrestins in apoptotic processes.

It is known that mitochondria represent the major intracellular source of ROS. β-arrestins are able to regulate mitochondrial ROS production (Philip et al., 2015). In cultured human cardiac fibroblasts isolated from failing hearts, there is an upregulation of β-arrestins that is associated with the activation of ERK and subsequent upregulation of Nox4, an NADPH oxidase able to catalyze the production of a superoxide free radical. This increase in oxidative stress promotes collagen synthesis and leads to myocardial fibrosis (Tsutsui et al., 2011). The overexpression of β-arrestins by means of adenoviral-mediated gene transfer increases mitochondrial superoxide production while the knockdown decreased ROS production and Nox4 expression in failing cardiac fibroblasts (Philip et al., 2015). Thus, targeted inhibition of β-arrestins in cardiac fibroblasts could be an effective strategy to decrease oxidative stress and fibrosis in cardiac tissue. Both β-arrestin 1 and β-arrestin 2 are both expressed in cardiac myocytes but it has been shown that only β-arrestin 1 is involved in βAR induced ROS production (J. Zhang et al., 2017). Indeed, the knockdown of β-arrestin1 inhibited βAR dependent mitochondrial ROS production while the knockdown of β-arrestin 2 exerted no effects. The mitochondrial production of ROS in response to βAR stimulation is activated by two different pathways at different times. Indeed, this study shows that the cAMP/PKA pathway is responsible for faster mitochondrial ROS production, whereas β-arrestin1 signaling is responsible for the slower one (Zhang et al., 2017).

Among β-arrestins interactome, many proteins participate to mitochondrial respiration (Gu et al., 2015), including metabolic enzymes involved in the glycolysis pathway (PK3, GAPDH, and enolase) and oxidative phosphorylation (ATP synthases and SDH2) (Xiao et al., 2007). The functional implications of such interactions are still unknown but likely β-arrestins interactions with these enzymes could be needed to promote energy production.

**FIGURE 3:**
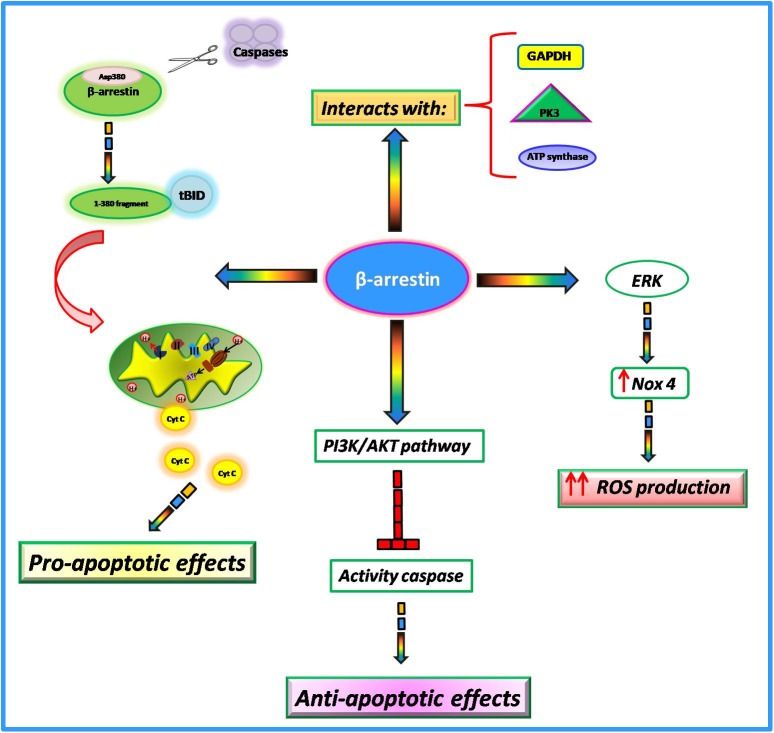
Non-GPCR activities of β-arrestins.

Compared with GRK2, the involvement of β-arrestins in the regulation of mitochondrial function needs more clarifications. However, the available findings suggest thatβ-arrestins interfere with several intracellular signaling pathways which are involved in the regulation of key mitochondrial processes (Figure 3) and could be a promising target for the regulation of mitochondrial function.

## Conclusion

Several reports support the proof of concept that GRK2 and β-arrestins are able to regulate intracellular signaling in a GPCR independent manner. These activities affect several compartments within the cell, including mitochondria. The involvement of GRK2 in the regulation of mitochondrial function has been recently identified showing its ability to regulate ATP content, ROS production, mitochondrial dynamics, and apoptosis. However, the exact role of the kinase (detrimental or protective) still remains to be elucidated given the opposing results from reports on this issue. Overall, we tried to reconcile these opposing findings pointing to a protective role of GRK2 in mitochondria through its binding to HSP90. Such an effect has important implications in the onset of cardiovascular disease, which are characterized by an impaired mitochondrial function. In this context, β-arrestins are novel identified targets whose activities in mitochondria are not completely clear yet. Few studies are available on β-arrestins dependent regulation of mitochondrial functions thus further investigations are needed. However, the available ones strongly suggest the involvement of β-arrestins in the regulation of mitochondrial ROS production and mitochondrial respiration. The better understanding of the role of these proteins in mitochondria could have important implications providing the basis for new therapeutic approaches to treat mitochondrial dysfunction in cardiovascular diseases.

## Author Contributions

DS and GI conceived the study. DS, JG, AF, GI, and MI wrote and critically reviewed the manuscript.

## Conflict of Interest Statement

The authors declare that the research was conducted in the absence of any commercial or financial relationships that could be construed as a potential conflict of interest.
